# Perceptions of TB-HIV comorbidity among the Nomads in Adamawa State, Nigeria

**DOI:** 10.1186/s12889-024-18414-z

**Published:** 2024-05-01

**Authors:** Suraj Abdulkarim, Stephen John, Tomon Garba, Hunpiya Basason, Paul Balogun, Joseph Kuye

**Affiliations:** 1 SUFABEL Community Development Initiative, Gombe, Gombe State Nigeria; 2 Janna Health Foundation, Yola, Adamawa State Nigeria; 3 John Snow Inc. (JSI), TB DIAH Project, Abuja, Nigeria

**Keywords:** Human Immunodeficiency Virus (HIV), Perception, Nomads, Mycobacterium Tuberculosis (MTB), Adamawa

## Abstract

The recalcitrance of Mycobacterium tuberculosis (MTB) to eradication was related to achieving a nonreplicating (dormant) state and the increasing global burden of HIV coinfection. Consequently, understanding the knowledge and perception of the population at risk of tuberculosis-HIV infection is essential to designing a strategy of intervention embraced by the target population. A cross-sectional study was conducted among Nomads in Adamawa State, Nigeria. A multistage sampling technique was employed to recruit consented participants. Self-administered questionnaires were used to gather the required information from 4 nomadic schoolteachers in each selected school. Data were entered into a Microsoft Excel sheet where trends and tables of collated data were developed. The findings show that only 13.5% of the participants expressed the correct perceptions of the complementary relationship between HIV and TB. More people in government employment (35%) understand the coexisting relationship of TB-HIV infections. At the same time, cattle herders and crop farmers who practice the prevalent occupation lack knowledge of TB-HIV relatedness. Across gender, only a proportion of males (14.8%) than females (10.5%) were more likely to show an understanding of the complementary association of HIV and TB, and this difference showed statistical significance (*p* = 0.0001). In conclusion, male gender, education at a degree or professional level, and employment with the government are factors associated with positive perceptions of TB/HIV relatedness. Thus, there is a need to intensify communication to educate Nomads on HIV and TB-related issues.

**Supplementary Information:**

The online version contains supplementary material available at 10.1186/s12889-024-18414-z.

## Introduction

Tuberculosis (TB) is an air-borne infectious disease caused by the *Mycobacterium tuberculosis complex.* It usually attacks the lungs and can spread to other body parts, such as the kidneys, spine, and brain [1]. More than 2 billion people are latently infected with TB, while an estimated 35.3 million people live with human immunodeficiency virus (HIV), 70% of whom live in sub-Saharan Africa [2–4]. According to the World Health Organization (WHO), 2017 an estimated 10 million people worldwide developed TB, while 1.3 million people died from the disease, including 0.3 million people living with HIV [5].

Despite concerted efforts and resources directed at managing TB across the globe, the recalcitrance of *Mycobacterium tuberculosis* (MTB) to eradication has not only resulted from it achieving a nonreplicating (dormant) state in the host [6], but its increasing global burden has also been linked to coinfection with HIV [6]. HIV infection-causing acquired immunodeficiency syndrome (AIDS) is one of the world’s most severe health and development challenges and a potent risk factor for TB [7]. Not only does HIV increase the risk of reactivating latent Mycobacterium tuberculosis infection, but it also increases the risk of rapid TB progression after infection or reinfection with Mycobacterium tuberculosis [6, 7], including resistant strains [8]. Up to 70% of patients with sputum smear-positive pulmonary TB observed in some sub-Saharan African countries were also HIV-positive, while TB also accounts for up to one-third of HIV/AIDS deaths worldwide [3, 9].

The most vulnerable to TB are women, children, and those with HIV/AIDS [10]. However, nomadic populations are especially prone to TB and HIV/AIDS infections. Nomads are communities of people who constantly migrate in search of pasture for their livestock, subsisting on hunting and gathering or often driven by climatic conditions [11, 12]. The nomadic population is often isolated and is socially vulnerable to diseases such as TB and HIV infections. They are characterized by economic poverty, relatively little access to health services, increased morbidity and mortality, remote geographical location, social exclusion, and migration, among other factors [12–14].

Nigeria, a country with one of the highest global TB burdens, is facing a challenge with low TB case detection rates. Only 16% of the estimated incident TB cases are currently being reported [15]. Adamawa State, with an estimated population of 3.7 million, including approximately 450,000 nomadic pastoralists (12% of the population), ranks among the eight states with the highest total TB notifications despite being only the twenty-seventh most populous state in 2010 [16]. Nomadic pastoralists often have limited access to healthcare, including TB services and immunization [17, 18]. The TB burden among nomadic pastoralists is worsened by factors such as low vaccination coverage (including the Bacille Calmette-Guérin vaccine), high rates of bovine TB, frequent consumption of unpasteurized milk, high levels of malnutrition, and living in poorly ventilated and overcrowded dwellings or tents [19, 20]. Additionally, nomadic populations tend to consult traditional healers, leading to delays in seeking care at health facilities [21].

In Adamawa State, Nigeria, where both tuberculosis (TB) and human immunodeficiency virus (HIV) have significant burdens, the comorbidity of TB and HIV is a critical concern, especially among nomadic populations. According to the Nigeria HIV/AIDS Indicator and Impact Survey (NAIIS) conducted in 2018, Adamawa State has a prevalence rate of 1.8% among adults aged 15–49 years, which is higher than the national average of 1.4%. This prevalence rate indicates that a significant number of people in Adamawa State are living with HIV/AIDS [22]. Similarly, TB is a significant public health issue in Nigeria, with low case detection rates and challenges in providing access to TB services, particularly among nomadic pastoralists.

Moreover, nomadic populations may tend to consult traditional healers, leading to delays in seeking care at health facilities. This delay in seeking care can result in the progression of TB and HIV infections, making treatment more challenging and reducing the effectiveness of treatment outcomes. Furthermore, the nomadic lifestyle can also lead to social and economic factors that may contribute to TB-HIV comorbidities, such as poverty, limited education, and gender inequality.

Since HIV, followed by TB, are the two leading causes of death from infectious diseases worldwide, their mutual reinforcement poses a greater risk to vulnerable communities [2, 9, 15]. Therefore, understanding the knowledge and perception of the population at risk of TB-HIV comorbidity is essential for designing a strategic intervention approach that the targeted population would embrace. Therefore, this study investigated the perceptions of Nomads in Adamawa State, Nigeria, on TB-HIV comorbidity.

## Material and Methods

### Study design

A cross-sectional study of the knowledge, attitude, and preventive practices of TB among Nomads in Adamawa State was conducted. A multistage sampling technique was employed to recruit consented participants. Three Adamawa state's local government areas (LGAs) were randomly selected for this investigation. Three nomadic schools and three nomadic communities were randomly selected in each LGA: nine schools and nine communities. After obtaining informed consent from the participants, self-administered questionnaires were used to gather the required information.

### Study settings


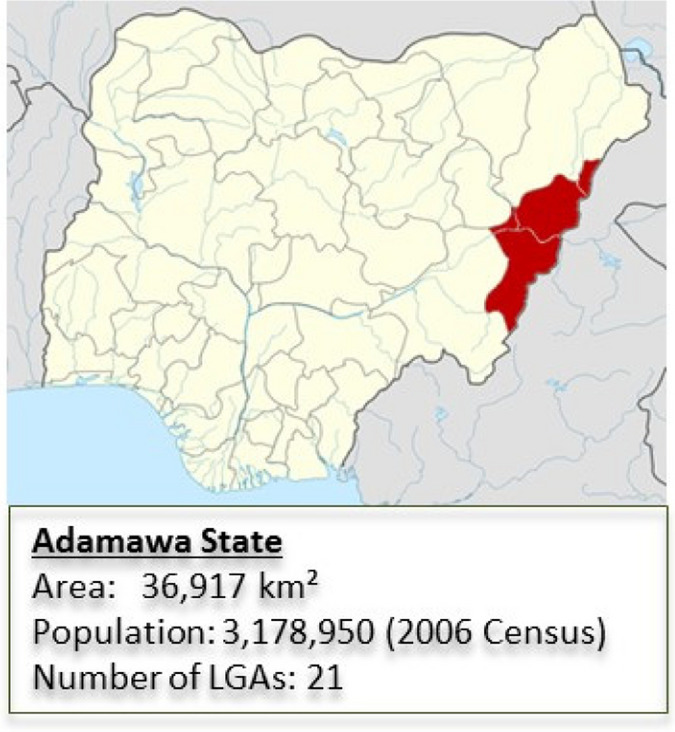


Adamawa is one of the largest states of Nigeria, located in the Northeast Geopolitical zone, with a population of 3,178,950 (2006 Census). Adamawa is bounded by Borno State to the north, Gombe and Taraba States to the north/west and south/west, respectively, and the Cameroons extending throughout its eastern border. Topographically, Adamawa has mountainous land crossed by large river valleys – Benue, Gongola, and Yedsarem. The valleys of the Cameroon, Mandara, and Adamawa mountains form part of the landscape. The state is administratively divided into 21 LGAs with elected councils in place. It has 25 legislative constituencies and three senatorial zones. Poverty levels in the state are reported to be among the highest in the country. The predominant economic activities in the state are subsistence farming and animal husbandry, which account for the livelihood of approximately 90% of the population. Their cash crops are cotton and groundnuts, while food crops include maize, yam, cassava, guinea corn, millet, and rice. The village communities living on the banks of the rivers engage in fishing, while the Fulanis are cattle rearers. The state has a network of roads linking all parts of the country. Adamawa State is one of the three states most affected by the insurgency in the north-eastern part of Nigeria. However, since mid-2015, the state has experienced relative calm. According to the IOM Displacement Tracking Matrix (DTM), as of October 2019, Adamawa has an estimated IDP population of 135,605. As such, a considerable number of displaced people are impacted by the conflict.

According to available data from the National Bureau of Statistics, Nigeria has 142 nomadic primary schools in Adamawa State as of 2019. This was the seventh-highest number among the 36 states of Nigeria [23]. Nomadic schools are educational institutions designed to cater to the needs of nomadic or semi-nomadic populations, such as pastoralists, who frequently move from one place to another. These schools are established to educate children who may otherwise be unable to attend traditional schools due to their mobile lifestyle. The curriculum may be tailored to the nomadic community's needs and cultural context. Health service provision in Adamawa state involves a wide range of public and private health service providers, including public health facilities managed by federal, state, and local governments, private for-profit providers, NGOs, community-based and faith-based organizations, and religious and traditional caregivers. The state has over 1,290 health facilities, including one federal medical centre, six general hospitals, one dermatological clinic, and eight cottage hospitals. The remaining are clinics, dispensaries, and health posts. Over 1,160 facilities are public, while 137 are private (12%). Available data show that Adamawa state has a healthcare worker (HCW) population density ratio well below the national average. Doctors/population and nurses and midwives/population ratios in Adamawa State were 1:29,840 and 1:7,197, respectively, above the World Health Organization’s recommendation of 1:10,000 and 1:5,000 [24]. The challenges the state faces include healthcare worker shortages, disparity in the distribution of health professionals between urban and rural deprived locations, and underutilization of skilled health workers, mainly due to weak systems and structures to effectively plan, manage, and develop the health workforce.

The study area has a total of 628 health facilities (public and private). Of these facilities, 109 provide DOTS services, 32 provide AFB sputum diagnostic and follow-up services, and 4 provide GeneXpert MTB/RIF testing services. Each treatment and diagnosis unit uses the NTBLCP standard TB recording and reporting tools (registers and forms) for TB case management and reporting.

Nomadic schools, also known as nomadic education, are a form of educational institution designed to cater to the needs of nomadic populations. These schools are established to educate children from nomadic or semi-nomadic backgrounds.

Nomadic schools are designed to be flexible and adaptable to the nomadic lifestyle. They often have mobile or semi-permanent structures, and the curriculum may be tailored to the nomadic community's specific needs and cultural context. Additionally, they may incorporate practical skills and knowledge relevant to the nomadic lifestyle, such as animal husbandry, traditional crafts, or sustainable agriculture.

The goal of nomadic education is to provide access to quality education for children from nomadic backgrounds, empowering them with the knowledge and skills needed to improve their livelihoods and contribute to the development of their communities.

### Study population

The study population encompasses adult nomadic schoolteachers and critical informants from nomadic communities, ensuring gender diversity in representation. Critical informants, comprising community leaders and community members, play a pivotal role in the study, as they possess in-depth knowledge and insights into the nomadic lifestyle, culture, and community dynamics. As part of the community, they are often more aware of the challenges and perceptions regarding TB-HIV comorbidity among nomads. By including these critical informants, the study aims to comprehensively understand the perceptions towards TB-HIV comorbidity within the nomadic population in Adamawa State, Nigeria.

### Sampling technique

A multistage sampling technique was employed to recruit consented participants. The study population included three local government areas (LGAs) in Adamawa state. A list of all the nomadic schools and communities was created at each LGA. The schools and communities were then assigned a unique number. A random number was generated manually using a table of random numbers. The random number corresponded to the first nomadic school/community selected. The next random number was generated to select the second nomadic school/community, and so on, until the required number of schools and communities were selected. Once selected, the schools and communities were visited. The sampling process included 36 nomadic teachers, 18 community leaders, and 27 community members. Four teachers from each nomadic school participated by using a self-administered questionnaire. Additionally, five key informants, including two leaders and three community members, were interviewed from each selected nomadic community to make up 81 respondents. The sampling technique involved random selection at each stage to ensure that the selection was unbiased and that any school or community had an equal chance of being selected.

### Data collection instrument

Data was collected through a structured questionnaire to gather demographic information and explore various facets of TB and HIV awareness, perceptions, and understanding. This encompassed the diseases' seriousness, curability, and sources of information. For questions regarding HIV and TB knowledge and curability, respondents could answer with "YES," "NO," or "Don't know." For questions on the seriousness of these diseases, respondents could choose from options such as "very serious," "somewhat serious," "not very serious," or "other." The survey was carefully crafted to provide a comprehensive view of the participants' insights and perspectives on TB and HIV.

### Interview procedure

Six interviewers were recruited and trained through a face-to-face interview approach. Interviews were conducted at the school and household levels. The participants were allowed to choose locations and languages that were convenient for them, but the analysis was conducted in English. Fieldwork supervision to ensure that privacy, confidentiality, interviewing techniques, and sampling methodologies were adhered to was provided by the Janna Health Foundation (JHF) team through spot checks during the study.

### Pretesting

The developed questionnaires were pretested by conducting 10 to 15 mock interviews with target group members in a neighbouring state. The pre-test was used to gather information on the ease or difficulty of the statements, comprehension, confidence in response, level of discomfort, and social desirability. Reliability of the questionnaire was also tested and a Cronbach Alpha value of 0.8 was obtained indicated high reliability.

### Data analysis

Data quality was continually monitored from the stage where data collectors were recruited until the database was complete and ready for analysis. Training and pretesting of tools were conducted to ensure consistency and compliance with the study protocol. Quantitative data collected through questionnaires were coded and entered into a Microsoft Excel sheet where trends and tables of collated data were developed. Data were exported to SPSS statistical software for advanced statistical analysis. Frequency counts, percentage, mean and Chi-square test were used to analyse the data. The findings were triangulated, while descriptive and inferential analyses of the trends and tables were presented. The responses were further categorized to better understand the study participants' knowledge level. Any score less than 60% of the maximum obtainable score is regarded as poor for knowledge and negative for perception. In comparison, scores of 60% and above are regarded as good for knowledge and positive for perception.

### Ethical considerations

Ethical clearance was sought, and approval from the Adamawa State Ministry of Health was obtained with approval number ADHREC /ADM/2018/071 and reference number S/MoH/1131/I. Written informed consent was obtained from all participants, and only the consented participants were recruited to participate in the study. For participants under 16 years, written informed consent was obtained from their parents and/or legal guardians. This study did not pose any physical risks associated with a physical procedure or intervention, such as obtaining tissue or blood samples. Nonetheless, the study was guided by principles and standards of the 2013 World Medical Association’s (WMA) version of Helsinki’s Declaration adoption on Medical Research Involving Human Subjects. Also, the protection of participants’ identity and autonomy, respect for their persons, and freedom to withdraw from participating in the interview was guaranteed.

## Results

The nomadic teachers expressed better knowledge of HIV (72.2%) than the community leaders (44.4%) and members (55.6%). Health workers (43.2%), followed by media broadcast (24.7%), were the main sources of HIV knowledge. Among nomadic teachers, 55.6% have heard of TB, while the respective percentages for community leaders and members are 50.0% and 51.9%. Notably, health workers are the primary source of TB knowledge for all groups, with the highest percentage among nomadic teachers (55.6%), followed by community members (33.3%) and community leaders (27.8%). Media broadcasts are also a significant source of TB knowledge, with 27.8% of nomadic teachers, 14.8% of community members, and 11.1% of community leaders citing this as their information source. According to 38.3% of the community members and 36% of Nomadic Teachers, HIV and TB were considered unrelated; 18.5% of community members and 13.3% of Nomadic Teachers perceived that both are the same, while on average, 13.6% of all respondents claimed that HIV and AIDS complement each other (Table 1).

**Table 1:** Patients' knowledge of tuberculosis and HIV

Parameter	Nomadic Teachers (%)	Community leaders (%)	Community members (%)	Total (%)
**Ever heard of HIV**				
Yes	26 (72.2)	8 (44.4)	15 (55.6)	49 (60.5)
No	4 (11.1)	3 (16.7)	5 (18.5)	12 (14.8)
No response	6 (16.7)	7 (38.9)	7 (25.9)	20 (24.7)
Total	36 (100)	18 (100)	27 (100)	81 (100)
**Ever heard of TB**				
Yes	20 (55.6)	9 (50.0)	14 (51.9)	43 (53.1)
No	7 (19.4)	2 (11.1)	6 (22.2)	15 (18.5)
No response	9 (25.0)	7 (38.9)	7 (25.9)	23 (28.4)
Total	36 (100)	18 (100)	27 (100)	81 (100)
**Source of knowledge of HIV**				
Religious Leaders	0 (0)	1 (5.6)	3 (11.1)	4 (4.9)
Health Workers	18 (50)	7 (38.9)	10 (37)	35 (43.2)
Family and Friends	1 (2.8)	4 (22.2)	2 (7.4)	7 (8.6)
Teachers	1 (2.8)	2 (11.1)	3 (11.1)	6 (7.4)
Posters and printed materials	0 (0)	0 (0)	1 (3.7)	1 (1.2)
Media (broadcasts)	12 (33.3)	4 (22.2)	4 (14.8)	20 (24.7)
Others	4 (11.1)	0 (0)	4 (14.8)	8 (9.9)
Total	36 (100)	18 (100)	27 (100)	81 (100)
**Source of knowledge of TB**				
Religious Leaders	1(2.8)	3(16.7)	4(14.8)	8(9.9)
Health Workers	20(55.6)	5(27.8)	9(33.3)	34(42.0)
Family and Friends	1(2.8)	5(27.8)	4(14.8)	10(12.3)
Teachers	2(5.6)	3(16.7)	2(7.4)	7(8.6)
Posters and printed materials	0(0)	0(0)	0(0)	0(0)
Media (broadcasts)	10(27.8)	2(11.1)	4(14.8)	16(19.8)
Others	2(5.6)	0(0)	4(14.8)	6(7.4)
Total	36 (100)	18 (100)	27 (100)	81 (100)
**The relationship of HIV with TB**				
They are the same	5 (13.9)	4 (22.2)	6 (22.2)	15 (18.5)
They are not related	13 (36.1)	9 (50)	9 (33.3)	31 (38.3)
They complement each other	8 (22.2)	3 (16.7)	0 (0)	11 (13.6)
I don’t know	4 (11.1)	0 (0)	4 (14.8)	8 (9.9)
Others	6 (16.7)	2 (11.1)	8 (29.6)	16 (19.8)
Total	36 (100)	18 (100)	27 (100)	81 (100)

**Fig. 1:**
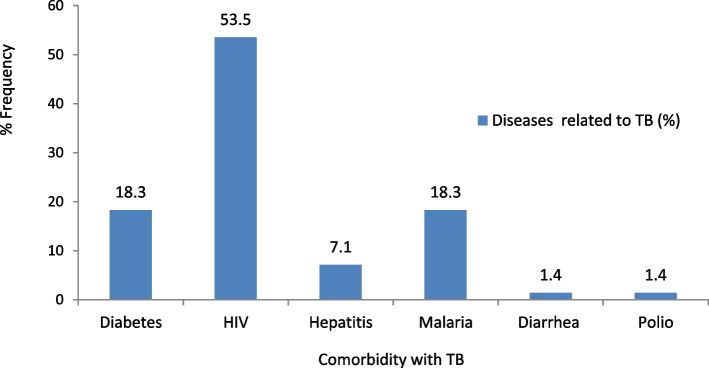
Percentage frequency of comorbidity of TB with other diseases (*n* = 71)

HIV was the most reported comorbidity with TB (53.59%), followed by diabetes (18.3%) and malaria (18.3%) (Fig. 1).

More than 55% of the participants perceived HIV as very serious, although 24.7% considered it not very serious. In their community, however, 45.7% admitted that HIV constitutes a severe disease, while more than half of the participants claimed that HIV cannot be cured, and 34.6% believed that the disease is curable. Regarding the seriousness of TB, 55.6% of nomadic teachers, 50.0% of community leaders, and 44.4% of community members consider TB to be very serious. The opinion of how serious TB is in the community closely reflects individual perceptions, with 47.2% of nomadic teachers, 44.4% of community leaders, and 33.3% of community members regarding TB as very serious. Notably, a higher proportion of community members (33.3%) perceive TB as not very serious compared to nomadic teachers (13.7%) and community leaders (22.2%). Concerning the curability of TB, the data show mixed opinions. While 33.3% of nomadic teachers believe TB can be cured, only 14.8% of community members hold this view. In contrast, 61.1% of nomadic teachers and 59.3% of community members do not think a person with TB can be cured (Table 2).

**Table 2:** Participants’ understanding of the prevalence of TB and HIV in the community

Parameter	Teachers (%)	Community leaders (%)	Community members (%)	Total (%)
**Opinion of how serious HIV is**				
Very Serious	22 (61.1)	8 (44.4)	15 (55.6)	45 (55.6)
Somewhat Serious	8 (22.2)	2 (11.1)	2 (7.4)	12 (14.8)
Not Very Serious	6 (16.7)	7 (38.9)	7 (25.9)	20 (24.7)
Others	0 (0)	1 (5.6)	3 (11.1)	4 (4.9)
Total	36 (100)	18 (100)	27 (100)	81 (100)
**Opinion of how serious HIV is in the community**				
Very Serious	18 (50)	8 (44.4)	11 (40.7)	37 (45.7)
Somewhat Serious	6 (16.7)	4 (22.2)	6 (22.2)	16 (19.8)
Not Very Serious	8 (22.2)	6 (33.3)	9 (33.3)	23 (28.4)
Others	4 (11.1))	0 (0)	1 (3.7)	5 (6.17)
Total	36 (100)	18 (100)	27 (100)	81 (100)
**Opinion on if person with HIV can be cured**				
Yes	17 (47.2)	7 (38.9)	4 (14.8)	28 (34.6)
No	16 (44.4)	10 (55.6)	17 (63)	43 (53.1)
No response	3 (8.3)	1 (5.6)	6 (22.2)	10 (12.3)
Total	36 (100)	18 (100)	27 (100)	81 (100)
**Opinion of how serious TB is**				
Very Serious	20 (55.6)	9 (50.0)	12 (44.4)	41 (50.6)
Somewhat Serious	10(27.8)	3 (16.7)	5 (18.5)	18 (22.2)
Not Very Serious	5 (13.7)	6 (33.3)	4 (14.8)	15 (18.6)
Others	1 (2.8)	0 (0)	6 (22.3)	7 (8.6)
Total	36 (100)	18 (100)	27 (100)	81 (100)
**Opinion of how serious TB is in the community**				
Very Serious	17 (47.2)	8 (44.4)	9 (33.3)	34 (42.0)
Somewhat Serious	9 (25.0)	6 (33.3)	8 (29.6)	23 (28.4)
Not Very Serious	6 (16.7)	4 (22.2)	9 (33.3)	19 (23.4)
Others	4 (11.1))	0 (0)	1 (3.7)	5(6.2)
Total	36 (100)	18 (100)	27 (100)	81 (100)
**Opinion on if person with TB can be cured**				
Yes	12 (33.3)	10 (55.6)	4 (14.8)	26(32.1)
No	22 (61.1)	8 (44.4)	16 (59.3)	46(56.8)
No response	2 (5.6)	0 (0)	7 (25.9)	7 (12.1)
Total	36 (100)	18 (100)	27 (100)	81 (100)

The association between the sociodemographic variables and participants’ perception of the relationship between HIV and TB showed no significant (*p* > 0.05) association with age. Across genders, only 14.8% of males were more likely to demonstrate an understanding of the complementary association of HIV and TB compared to 10.5% of females, and the association was statistically significant (*p* = 0.0001). Additionally, a higher proportion of people educated at the professional or degree level (62.2%) perceived that HIV-TB complements each other; education level contributed significantly (*p* = 0.001) to participants' knowledge of TB-HIV comorbidity. Similarly, participants in government employment (35%) were those who showed an understanding of the HIV-TB relationship, and this showed a statistically significant (*p* < 0.0001) association (Table 3).

**Table 3:** Association between participants’ sociodemographic characteristics and their perception of the relatedness of HIV and TB

Parameters	Variables	Perception of the association between HIV and TB						Inferential analysis		
		**Same %**	**Not related %**	**Complement each other %**	**Don’t know %**	**Others %**	**Total%**	**df**	$\boldsymbol x^{\mathbf2}$	***P*** **-value**
**Age**	15–24	1(14.3)	2(28.6)	0(0)	0(0)	4(57.1)	7(100)	20	30.34	0.065
	25–34	9(47.4)	6(31.2)	1(5.3)	1(5.3)	2(10.5)	19(100)			
	35–44	3(12.5)	9(37.5)	3(12.5)	3(12.5)	6(25)	24(100)			
	45–54	2(10)	9(45)	5(25)	3(15)	1(5)	20(100)			
	55–64	0(0)	3(42.9)	2(28.6)	0(0)	2(28.6)	7(100)			
	65 and above	0(0)	2(50)	0(0)	1(25)	1(25)	4(100)			
	Total	15(18.5)	31(38.3)	11(13.8)	8(9.9)	16(19.8)	81(100)			
**Gender**	Male	14(22.6)	29(46.8)	9(14.5)	6(9.7)	4(6.5)	62(100)	4	26.85	0.0001 ** f
	Female	1(5.3)	2(10.5)	2(10.5)	2(10.5)	12(63.2)	19(100)			
	Total	15(18.5)	31(38.3)	11(13.6)	8(9.9)	16(19.8)	81(100)			
**Education**	No school at all	1(5.6)	7(38.9)	2(11.1)	3(16.7)	5(27.8)	18(100)	52.73	24	0.001**
	Literacy classes only	5(33.3)	7(46.7)	0(0)	3(20)	0(0)	15(100)			
	Some primary school	3(17.6)	8(47.1)	1(5.9)	2(11.8)	3(17.6)	17(100)			
	Completed primary school	0(0)	1(16.7)	0(0)	0(0)	5(83.3)	6(100)			
	Some high school	2(25)	4(50)	2(25)	0(0)	0(0)	8(100)			
	Completed high school	3(33.3)	4(44.4)	1(11.1)	0(0)	1(11.1)	9(100)			
	Professional or Degree	1(12.5)	0(0)	5(62.5)	0(0)	2(25)	8(100)			
	Total	15(18.5)	31(38.3)	11(13.6)	8(9.9)	16(19.8)	81(100)			
**Work**	Cattle rearing	4(20)	12(60)	1(5)	2(10)	1(5)	20(100)	35.38	12	0.0001**
	Crop Farming	3(13)	11(47.8)	1(4.3)	4(17.4)	4(17.4)	23(100)			
	Government Employment	5(25)	6(30)	7(35)	1(5)	1(5)	20(100)			
	Others	3(16.7)	2(11.1)	2(11.1)	1(5.6)	10(55.6)	18(100)			
	Total	15(18.5)	31(38.3)	11(13.6)	8(9.9)	16(19.8)	81(100)			

f = Fisher’s exact test. Significant: *p* ≤ 0.05 = *, *p* ≤ 0.01 = **

The data of the three sub-populations were consolidated for analysis to capture a holistic view of the nomadic community's perceptions. Despite their roles (teachers, leaders, community members), all individuals are part of the broader nomadic population, sharing common cultural, social, and economic traits that shape their views and behaviours regarding health issues like HIV and TB. This approach allows for a more inclusive understanding of the nomadic community's outlook. While teachers may have higher education levels and distinct roles, community leaders and members offer insights from diverse social strata and daily experiences. Maintaining separate datasets for each subgroup could result in underpowered subgroup analyses due to small sample sizes, potentially hindering the study's statistical robustness and reliability.

Table 4 reflects the knowledge and perception of HIV and TB among nomadic teachers, community leaders, and community members. Notably, a significant portion of each group (52.8% of teachers, 61.1% of leaders, and 40.7% of members) exhibits "good" knowledge of these diseases (Table 4). Conversely, "poor" knowledge is observed in 47.2% of teachers, 38.9% of leaders, and 59.3% of community members (Table 4). In terms of perception, the majority of all groups (94.4% of teachers, 88.9% of leaders, and 77.8% of members) have "positive" perceptions of HIV and TB (Table 4). However, a small proportion (5.6% of teachers, 11.1% of leaders, and 22.2% of members) hold "negative" perceptions (Table 4). This comprehensive overview suggests a varied understanding of these diseases among the different groups, highlighting potential areas for targeted education and awareness campaigns.

**Table 4:** Levels of Nomads Knowledge and Perception of HIV and TB

Parameter	Nomadic Teachers (%)	Community leaders (%)	Community members (%)	Total (%)
**Knowledge of HIV and TB**				
Good	19(52.8)	11(61.1)	11(40.7)	41(50.6)
Poor	17(47.2)	7(38.9)	16(59.3)	40(49.4)
Total	36 (100)	18 (100)	27 (100)	81 (100)
**Perception of HIV and TB**				
Positive	34(94.4)	16(88.9)	21(77.8)	71(87.7)
No	2(5.6)	2(11.1)	6(22.2)	10(12.3)
Total	36 (100)	18 (100)	27 (100)	81 (100)

## Discussion

Most of the participants had heard about HIV, possibly due to the high prevalence of the disease, especially in sub-Saharan Africa [16]. Nevertheless, the higher awareness rate among the nomadic teachers can be attributed to the better access to information and exposure they have gained, considering the nature of their occupation. A similar observation was attested to in a report that having knowledge and information is the first key and necessary element in developing health behaviour; thus, if teachers know about HIV/AIDS, they can transfer such knowledge and positive attitudes to their students [17].

Health workers, followed by media broadcasts, were the primary sources of HIV knowledge for the participants. This justifies the significant health resources invested in creating and utilizing various outlets to deliver HIV/AIDS information to the public. More so, television, radio, newspapers, periodicals, direct counselling from medical staff, and dispersion of information through local family and friend networks have been highlighted as sources of HIV/AIDS information [18]

The perception of many that HIV and TB are either the same or not related is contrary to the opinion shared in some earlier reports that HIV infection constitutes a potent risk factor for TB, and it increases the risk of reactivating latent Mycobacterium tuberculosis infection as well as increasing TB progression [6]. Only 13.6% of participants in this study expressed a similar view that HIV and TB complement each other. This was further validated by Raviglione Raviglione et al. [9], who considered HIV/AIDS the primary threat to TB control programmes in Africa since as HIV prevalence rises, so does TB, and TB rates will plateau once HIV infection does. Furthermore, TB comorbidities, including HIV, diabetes, hepatitis, and malaria, among others highlighted, affirmed the report of TB comorbidities [19]. HIV (53.5%) was the most prevalent comorbidity recorded in this study, followed by diabetes (18.3%) and malaria (18.3%). This further underscores the interaction between HIV and TB. Moreover, the increasing global burden of TB has been linked to HIV infections [6].

More than half of the participants who considered HIV a severe disease demonstrated their understanding of the aetiology of the infection. However, this knowledge has been considered a helpful instrument in managing the disease; thus, the theory of planned behaviour asserts that perceptions of HIV susceptibility severity drive HIV-negative individuals’ motivations to use HIV prevention methods [20, 21]. The seriousness of HIV in the nomads' community, rated below average by the participants, could be associated with the gap in knowledge concerning the burden of HIV infection among the nomadic Fulani of northern Nigeria, although migration, which is a way of life of this population, is known to increase the rate of HIV transmission and limits individuals’ access to treatment and care [22].

Generally, only 13.5% of the participants expressed the correct perceptions of the complementary relationship between HIV and TB. This reveals an enormous gap in the knowledge of the aetiology of TB among nomadic populations. Perception of this category was found to be in line with active TB disease being linked with the breakdown in immune surveillance; this explains the strong association between active TB disease and other infectious or noncommunicable diseases that exercise a toll on the immune system [25]. HIV is the most significant risk factor for activating latent tuberculosis infection [23].

Gender, education levels, and occupation, as the factors found to be significantly associated with participants’ perception of TB-HIV relatedness, affirm the significant influence of social factors on the health outcome of a community [24]. Male participants (14.5%) were revealed as having the correct perceptions of the females, which could possibly be explained by their role as decision-makers in the family [26]. Mainly, the participants at the degree or professional levels (62.5%) demonstrated good understanding, thus affirming the impact of education in receiving and synthesizing information [17]. Similarly, more people in government employment (35%) than other occupations understand the coexisting relationship of TB-HIV infections. In contrast, cattle rearers and crop farmers who practice prevalent occupations in the nomadic community lack knowledge of TB-HIV relatedness [6, 11, 12].

## Limitations

The study focused on a specific population of nomads in Adamawa State, Nigeria. This limits the generalizability of the results to other populations, particularly those in urban or non-nomadic rural settings. Another limitation is the potential for recall bias, especially in questions related to sources of information about TB and HIV. Participants may have had difficulty remembering specific sources, leading to inaccuracies in their responses. Similarly, one limitation of this study is the potential for response bias. Since the data was self-reported, there's a possibility that participants may have misreported their level of awareness, perceptions, and understanding of TB and HIV. This could lead to an overestimation or underestimation of these variables, affecting the accuracy of the findings.

## Conclusion

The study focuses on understanding the knowledge and perception of people at risk of TB-HIV comorbidity. Developing a strategy intervention approach to be adopted in any related project is essential. The study shows that knowledge gaps exist. Most participants erroneously recognized the complementary relationship between HIV and tuberculosis. Gender, higher education, and professional training, as well as employment in government organizations, are factors that have a positive perception of the link between tuberculosis and HIV. Cattle rearers and crop farmers in the nomadic community who practice the common occupation do not know the connection between TB and HIV.

## Recommendation

Based on the findings of this study, the following are recommended:

1. The results show that there is a gap in knowledge across genders. Relevant stakeholders and bodies concerned should promote awareness of HIV and TB through Community Health Influencers Promoters and Services (CHIPS), who encourage and support local households to seek healthcare services in various communities.
2. When advocating or briefing on HIV or TB interventions, key actors should encourage community representatives to share knowledge and support them in presenting information on the coexistence of TB-HIV infections, especially in communities where people are active in common occupations such as livestock and agricultural industries.
3. During interventions on tuberculosis and HIV, individuals from various community interventions should be involved. This will facilitate access and promote knowledge on the coexistence of TB-HIV infections among all target populations.
4. The government and key actors of related project intervention should sponsor monthly dissemination of information on media stations regarding tuberculosis and coinfection diseases. This will help reach out to many community members who use radio, TV, etc.

### Supplementary Information

**Supplementary Material 1.**

## Data Availability

All relevant data are within the paper and supporting information files. Additional information about the databases used for this study is available from Nigeria's National TB and Leprosy Control Program. www.ntblcp.org.ng/
